# A Novel Protocol for Detection of Senescence and Calcification Markers by Fluorescence Microscopy

**DOI:** 10.3390/ijms21103475

**Published:** 2020-05-14

**Authors:** Jaqueline Herrmann, Milen Babic, Markus Tölle, Kai-Uwe Eckardt, Markus van der Giet, Mirjam Schuchardt

**Affiliations:** 1 Department of Nephrology and Medical Intensive Care, Charité—Universitätsmedizin Berlin, Corporate Member of Freie Universität Berlin, Humboldt-Universtität zu Berlin, and Berlin Institute of Health, Hindenburgdamm 30, 12203 Berlin, Germany; Jaqueline.Herrmann@charite.de (J.H.); Milen.Babic@charite.de (M.B.); Markus.Toelle@charite.de (M.T.); Kai-Uwe.Eckardt@charite.de (K.-U.E.); Mirjam.Schuchardt@charite.de (M.S.); 2 Department of Chemistry, Biochemistry and Pharmacy, Freie Universität Berlin, Königin-Luise Straße 2+4, 14195 Berlin, Germany

**Keywords:** calcification, senescence, smooth muscle cell, SA-β-galactosidase, senescence-associated heterochromatin foci

## Abstract

Vascular calcification and stiffening of the arterial wall is a systemic phenomenon that is associated with aging and it can be increased by several risk factors. The underlying mechanisms, especially the pathways of cellular senescence, are under current investigation. Easily manageable in vitro settings help to study the signaling pathways. The experimental setting presented here is based on an in vitro model using rat vascular smooth muscle cells and the detection of senescence and osteoblastic markers via immunofluorescence and RNAscope™. Co-staining of the senescence marker p21, the osteoblastic marker osteopontin, detection of senescence-associated heterochromatin foci, and senescence-associated β-galactosidase is possible within one test approach requiring fewer cells. The protocol is a fast and reliable evaluation method for multiplexing of calcifying and senescence markers with fluorescence microscopy detection. The experimental setting enables analysis on single cell basis and allows detection of intra-individual variances of cultured cells.

## 1. Introduction

Aging is associated with a variety of characteristic changes of the vessel wall [1]. There are several disorders, in which patients show signs of premature aging of vessels that appear much older than their biological age e.g., in chronic kidney disease [2,3,4]. A hallmark of vascular aging is a stiffening of the arterial wall with increasing pulse-wave velocity and the mineralization of vascular smooth muscle cells (VSMC) in the media layer of the vessel wall [1,5]. Treatment options are currently not available [5].

Resulting from clinical and basic research, there is strong evidence that vascular calcification and aging occur jointly [4,6,7]. Several underlying mechanisms are discussed for vessel mineralization, including, but not limited to, oxidative stress from various sources and resulting DNA damage, continuous inflammation, and activation of pro-osteogenic signaling pathways [3,7,8]. However, the underlying pathophysiological mechanisms need further clarification. Currently, several hypotheses exist regarding joint or consecutive appearance of calcification and senescence in a vicious cycle in smooth muscle cells e.g., induced by uremic toxins [4,9]. In addition, the senescence level can vary within and between cells and the tissue of the same individual [10]. It has to be illuminated whether, in one cell population, cells experience senescence and calcification jointly or consecutively, or whether aged vessels contain distinctly different cell populations of aged and calcified cells.

A better understanding of the underlying mechanisms inducing and linking calcification and senescence of VSMC in the vessel wall will be necessary for establishing promising treatment options.

Detecting cellular senescence is hampered by the heterogeneity of senescence markers. For the reliable identification of DNA damage and cell senescence, the detection of several known markers is necessary. Here, the increased messenger-ribonucleic acid (mRNA) and protein expression of the cell cycle protein and cyclin inhibitor p21 as well as the formation of senescence-associated heterochromatin foci (SAHF) and the accumulation of senescence-associated β-galactosidase (SA-β-Gal) are typically used [10,11,12]. Additionally, the cells often undergo morphological changes that are detectable by light microscopy. Cell mineralization is accompanied by a shift in expression of a wide array of different markers e.g., osteopontin (OPN) [3,13].

The study aims to develop a fast, robust, and easy to handle protocol by detection of SA-β-Gal, SAHF, p21, OPN, and control of cell morphology, on single cell basis in vitro in order to facilitate jointly and/or consecutively activation of senescence and calcification markers within the cells in a parallel experimental setting. Therefore, the described method combines ultrasensitive RNAscope™ in situ hybridization and immunohistochemistry in a multiplex approach requiring few cells. Other currently available experimental settings with their advantages and disadvantages are summarized in the supplementary Table S1. Briefly, the in situ hybridization technique enables the detection of various nucleotide sequences in cells and tissue by radioactive, fluorescence, or immunohistochemistry labeling and it permits a multiplex approach in the right experimental setting [14]. The protocol presented here utilizes RNAscope™, a technology that applies oligonucleotide probes and immunohistochemical or fluorescence-based detection [15]. The utilized RNAscope™ kit allows for the analysis of three targets of interest in one experimental setting by fluorescence-based detection.

For the establishment and optimization of the protocol steps, the known inductor of cell senescence, doxorubicin (Dox) [16], as well as a known inducer of cell mineralization, the uremic toxin uridine-adenosine tetraphosphate (Up_4_A) [17], were used. The established protocol provides reliable data while requiring fewer primary cell numbers and, therefore, fewer animals per experiment by multiplexing several markers of interest.

## 2. Results and Discussion

Currently, several senescence markers for cells and tissue are known [10,11]. Often, the detection of more than one marker is used for reliable detection and the senescence levels can vary between cells and tissue, respectively, within the same animal [10].

Here, we provided a staining protocol for four markers of interest to detect senescence and osteoblastic differentiation in cells. Besides, the protocol can be expanded for the detection of other markers by using a laser-scanning microscope for image acquisition. The detection via fluorescence staining allows not only the visualization in individual cells, but also quantification for statistical analysis of the results.

### 2.1. Detection of SA-β-Gal

**Figure 1:**
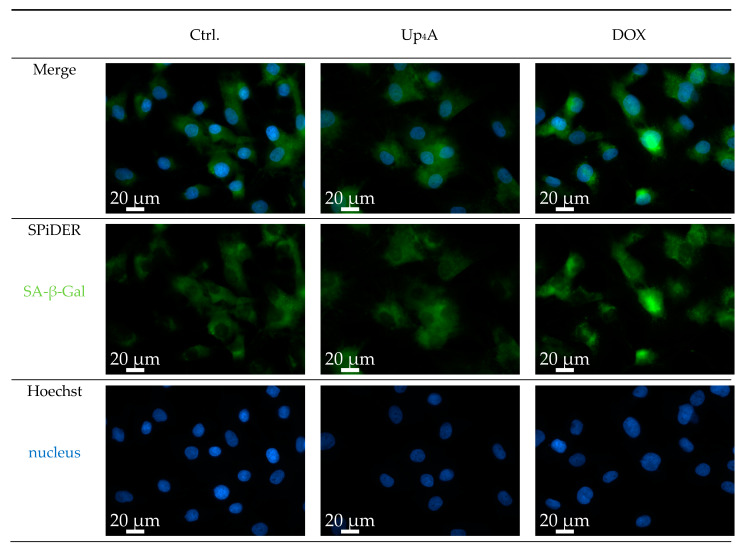
Detection of senescence-associated β-galactosidase (SA-β-Gal) in primary rat smooth muscle cells upon stimulation with doxorubicin (Dox, 500 nmol/L) and uridine-adenosine tetraphosphate (Up_4_A, 100 µmol/L) for 72 h. 40× objective. Representative images out of three independent experiments. Ctrl.: control.

**Figure 2:**
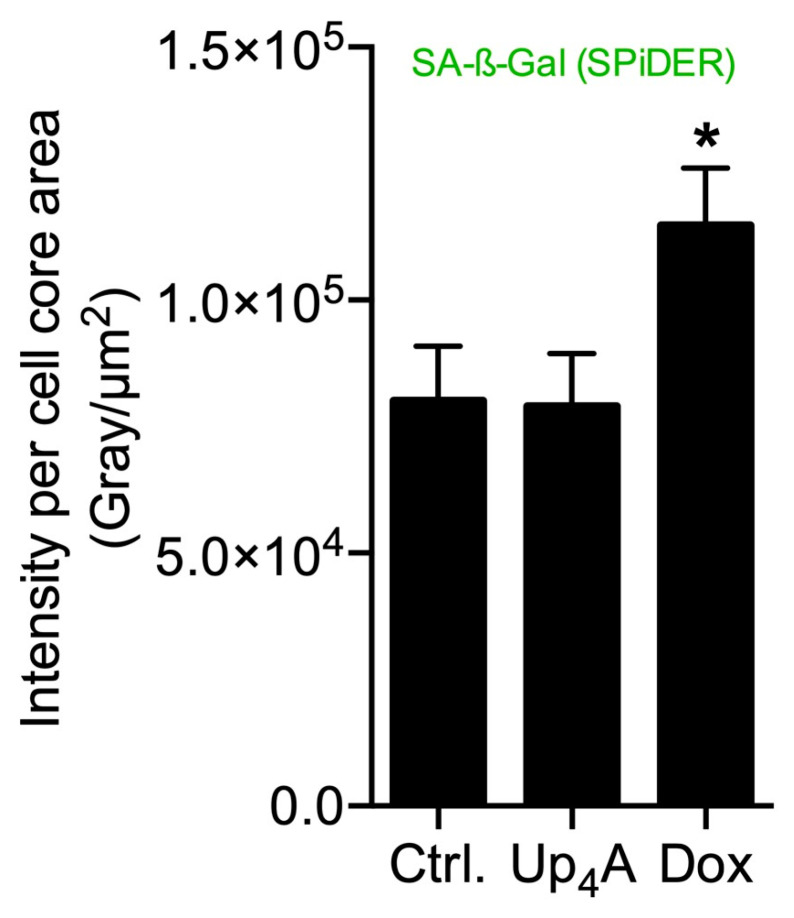
Quantification of senescence-associated β-galactosidase (SA-β-Gal) in primary rat smooth muscle cells upon stimulation with doxorubicin (Dox, 500 nmol/L) and uridine-adenosine tetraphosphate (Up_4_A, 100 µmol/L) for 72 h. For analysis, intensity pixel sum per channel was normalized to cell core area. Bar graph represents mean ± SEM of three independent experiments. Ctrl.: control.

SA-β-Gal was selected as marker accumulating in cells during aging, according to previous studies [6,10]. The staining of the cells with SPiDER-SA-ß-Gal upon stimulation with Dox, as a known inductor of cell senescence [16], results in the accumulation of SA-β-Gal within the cytoplasm of the cell, as shown in Figure 1. In contrast, while Up_4_A induces cell calcification [17], no accumulation of SA-β-Gal could be detected upon stimulation of VSMC for 72 h. For counterstaining of the nucleus, Hoechst stain was used. The quantification of the pixel sum intensity (Figure 2) confirmed the findings of the representative images that are shown in Figure 1.

The results suggest that accumulation of SA-β-Gal in rat vascular smooth muscle cells (rVSMC) depends on the inductor and may be dependent on time for different inductors. This underlines the fact that the detection of more than one senescence marker often seems necessary [10].

### 2.2. OPN and p21 mRNA Detection and Detection of SAHF

**Figure 3:**
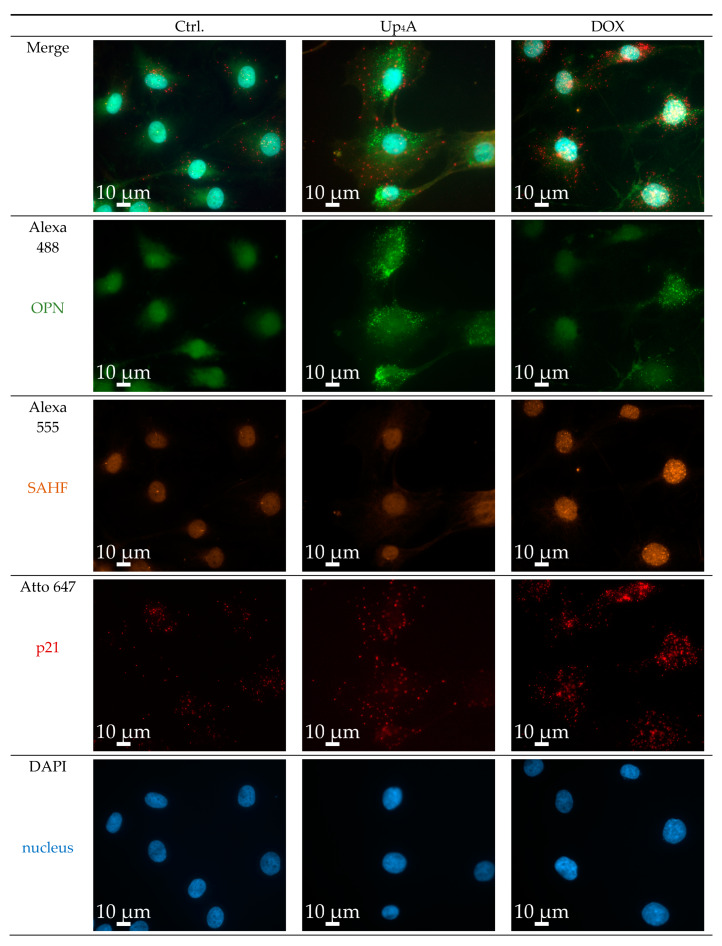
Detection of osteopontin (OPN), senescence-associated heterochromatin foci (SAHF), and p21 in primary rat smooth muscle cells upon stimulation with doxorubicin (Dox, 500 nmol/L) and uridine-adenosine tetraphosphate (Up_4_A, 100 µmol/L) for 48 h. 40× objective. Representative images out of three independent experiments. Ctrl.: control.

OPN was selected as a calcification marker, because its gene expression was increased by stimulation with different calcification inducers in previous experiments [17]. The gene expression of the cyclin inhibitor p21 is one typical marker used for the detection of cellular senescence [6,10]. Several experimental protocols currently exist for the detection of calcification or senescence markers in vascular cells (summarized in Table S1). However, the current goal was to use a single cell-based identification of a senescence and osteoblastic marker while using RNA in situ hybridization via RNAscope™ technology. The significant advantage is the multiplexing possibility of several target genes of interest in a cell and tissue sample in a robust way with high sensitivity [15]. Dox strongly induced SAHF and mRNA expression of p21, but only slightly induced OPN mRNA expression upon 48 h of stimulation, as shown in Figure 3. In contrast, upon Up_4_A stimulation, OPN mRNA expression is induced profoundly, but no SAHF could be detected and p21 mRNA expression is not induced in VSMC.

**Figure 4:**
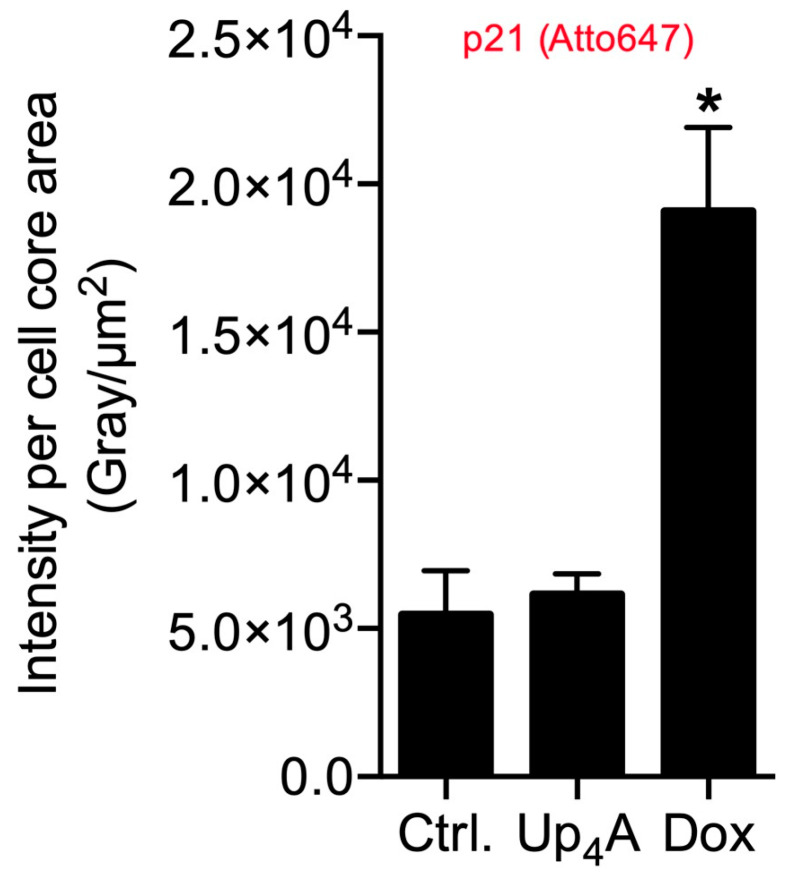
Quantification of p21 in primary rat smooth muscle cells upon stimulation with doxorubicin (Dox, 500 nmol/L) and uridine-adenosine tetraphosphate (Up_4_A, 100 µmol/L) for 48 h. For analysis, intensity pixel sum per channel was normalized to cell core area. Bar graph represents mean ± SEM of three independent experiments. Ctrl.: control.

The quantification of the pixel sum intensity confirmed the findings for channel Atto 647 (Figure 4). The crosstalk between the channels Alexa 488 and Alexa 555 and the resulting background impedes the quantification of OPN mRNA expression and SAHF formation with a standardized, automated, and reproducible ZEN protocol in this experimental setting. Several options exist to overcome this issue: next to the application of alternative software permitting manual identification of targets and subsequent quantification, which can be prone to researcher bias and, therefore, is not presented here, imaging with a confocal microscope could facilitate quantification by reducing cross talk and background.

Stimulation with different inducers as Dox and Up_4_A varies in the markers analyzed for calcification and senescence. This underlines that, often, the detection of more than one senescence and calcification marker seems to be necessary for reliable and comparable results. In addition, in our experimental setting, we observed a certain level of cell batch specific variances in the expression levels of markers that are comparable to alternative protocols, like Western Blot and polymerase chain reaction (PCR). However, representative images also illustrate differences in marker expression between individual cells, especially for the investigated calcification marker OPN. This information is critical in order to understand the mechanisms and pathways within one cell during the calcification process. This information is lost in protocols that jointly analyze cell bulks. The current protocol can allow for the analysis of co-localization analysis within one cell and differences between cells of the same cell batch.

### 2.3. Limitations

The experimental design that is presented here is established for an in vitro experiment. Nevertheless, a transfer from cells to tissue should be possible. The development of appropriate pre-treatment and imaging of tissue section will require further optimization steps. In the case of SA-β-Gal staining in tissue, we recommend using freshly frozen tissue and process the material immediately, as storage even at −80°C reduces enzyme activity. In a proof of concept experiment, we tested RNAscope™ staining in frozen and paraffin-embedded aortic sections (unpublished data). We found a similar background in both materials and better results in paraffin-embedded tissue, which we attribute to easier handling. Alternative targets might be of interest, according to the focus of research. Here, our protocol provides some opportunities for variation: alternative target genes can be analyzed with the RNA in situ hybridization technique. The utilized protocol allows up to three different target genes. We used one of the available channels for immune-histological staining to make use of the optimal capacity of our microscope. If equipped with alternative hardware, combination with another secondary antibody for immune histological staining is possible, thus enriching the opportunities for multiplexing.

We are aware that there is a variety of alternative protocols. Alternative research models, such as primary cells from mouse or human, cell lines, as well as tissue sections from clinical or laboratory origin, are suitable alternatives. Next to that, a huge variety of different methods for the detection and quantification of our selected targets are possible. Each comes with its advantages and disadvantages that are shortly summarized in Table S1. In comparison to 5-bromo-4-chloro-3-indolyl-β-D-galactopyranoside (X-Gal/BCIG) staining, we found fluorescence staining of SA-β-Gal to be more robust, faster, and easier to image.

## 3. Materials and Methods

**Figure 5:**
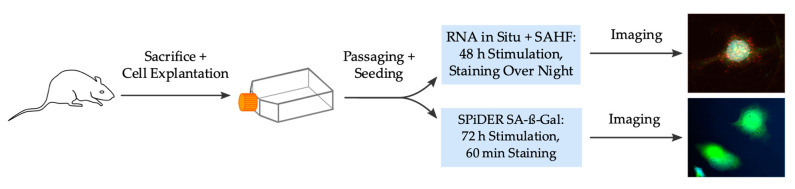
Scheme of the complete experimental workflow. SAHF: senescence-associated heterochromatin foci, SA-ß-Gal: senescence-associated ß-galactosidase.

Figure 5 summarizes the complete and stepwise workflow of the experimental procedures. Primary VSMC from rat thoracic aorta was selected for experiments. Further special ordering information for kits with its components and antibodies can be found in the Supplementary Material (Tables S2 and S3).

### 3.1. Cell Isolation and Culturing

The study was in accordance with the EU Directive 2010/63/EU for animal experiments and it was approved by the Landesamt für Gesundheit und Soziales Berlin, Germany (T0211/02) and the animal facility of the Charité—Universitätsmedizin Berlin, Germany. The aorta of Wistar rats was prepared after euthanasia with sodium pentobarbital (400 mg/kg body weight) per intraperitoneal injection. After removal of the adventitia of rat thoracic aorta, primary rat VSMC were isolated by explant outgrowth, as described previously [17]. VSMC were cultured in Dulbecco Modified Eagle Medium (DMEM, Biochrom AG) containing 1 g/L glucose, supplemented with 10% fetal calf serum (FCS, Biochrom AG), penicillin (100 U/mL, Biochrom AG), and streptomycin (0.1 mg/mL, Biochrom AG). The cells were cultured in a humidified incubator at 37°C with 5% carbon dioxide. Cells at passages 4 were used for experiments. The cells were seeded in IBIDI 8 Well µ- Slides (ibidi GmbH) for SA-ß-Gal staining and 8-well LabTec Chamber Slides (Thermo Scientific) for RNA in situ hybridization. Cells were cultured for 24 h to a confluence of 70–80%. It is essential to ensure subconfluence of cells prior stimulation, because confluence itself was described as an inducer of SA-β-Gal activity [18]. The cells were serum starved for 24 h prior stimulation in DMEM containing 4.5 g/L glucose, supplemented with 1% glutamin and antibiotics (penicillin 100 U/mL, streptomycin 0.1 mg/mL). This medium was also used for stimulation. For SA-β-Gal staining, the cells were stimulated for 72 h, whereas for mRNA and SAHF detection a stimulation time of 48 h was used.

### 3.2. Experimental Setting for Detection of SA-β-Galactosidase

Information regarding the kit components and ordering information are summarized in the Supplementary Material (Table S3).

#### 3.2.1. Preparations

- Heat incubator to 37 °C (no humidity and carbon dioxide control).
- Warm 4% formalin and PBS to 37 °C.
- Prepare McIlvaine buffer: Mix 7.4 mL 0.1 mol/L citric acid solution and 12.6 mL 0.2 mol/L sodium phosphate solution and set pH to 6.0.
- Solve 20 µg SPiDER-SA-β-Gal (Gerbu Biotechnologie) in 35 µL dimethylsulfoxide (DMSO). Store aliquots at −20°C.
- Dilute McIlvaine buffer 1:5 in ultrapure water and warm dilution to 37 °C. Dilute SPiDER-SA-ß-Gal 1:500 in McIlvaine buffer in order to obtain the working solution. Protect working solution from light.
- Prepare Hoechst working solution by dissolving Hoechst 33342 (Thermo Fisher) in the appropriate amount of water to obtain a stock concentration of 10 mg/mL. The stock concentration can be aliquoted and stored at −20°C. To obtain the working solution, dilute Hoechst 1:2000 in PBS. Protect working solution from light.

#### 3.2.2. Staining Procedure

**Figure 6:**
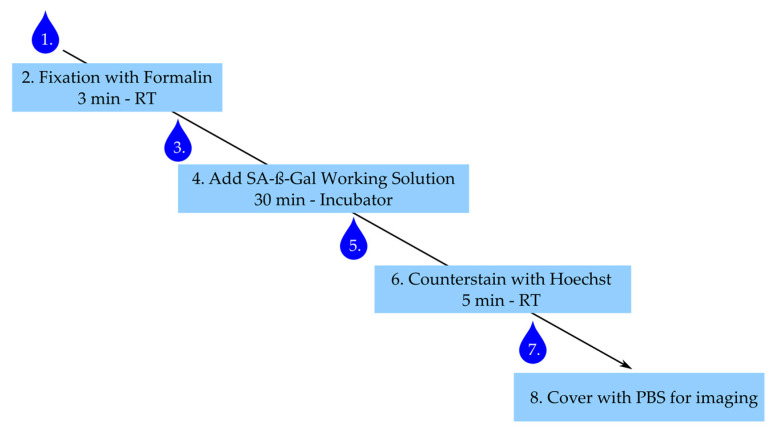
Staining procedure for senescence-associated-β-galactosidase (SA-ß-Gal). Numbering represents steps in staining procedure. Drop corresponds to wash step. RT: room temperature.

The step-by-step staining procedure according to the manufacturer’s (Dojindo) recommendations is given below. Figure 6 summarizes the main steps with an incubation time less than 40 min for the whole procedure.

1. After stimulation, aspirate medium and wash cells once with PBS.
2. Add 300 µL of 4% buffered formalin to each well and fix cells for 3 min at room temperature, ensure the exact fixation time.
3. Aspirate formalin and wash three times with warm PBS.
4. Add 300 µL of working solution per well and incubate for 30 min in the incubator under light protection. Caution: Ensure the right fixation times and pH conditions—this is critical for the β-Gal staining.
5. Aspirate working solution and wash cells twice with PBS.
6. Counterstain with Hoechst working solution for 5 min under light protection.
7. Aspirate solution and wash once with PBS.
8. Add 300 µL of PBS and image within 24 h.

### 3.3. Experimental Setting for Detection of OPN, p21 and SAHF

All kit components for staining with the ordering information are summarized in the Supplementary Material (Table S3).

#### 3.3.1. Preparations

- Add purified water to the humidity control tray and heat oven to 40 °C.
- Thaw ProLong™ diamond antifade medium (Thermo Scientific) at room temperature.
- Prepare wash buffer (ACD Bio) according to manufacturer’s instructions.
- Heat mRNA probes (ACD Bio) gently at 40 °C for 10 min in a water bath, centrifuge probes and mix according to manufacturer’s instructions.
- Dilute Protease III (ACD Bio) 1:15 with PBS.
- Prepare washing dishes for PBS and wash buffer.
- Prepare 10% Roti™ImmunoBlock (Carl Roth) by diluting in PBS.
- Prepare 1:500 dilution of primary antibody anti-histone H2A.X (phosphoS139) antibody [EP854(2)Y] (abcam) in 1% Roti™ImmunoBlock/PBS.
- Prepare 1:1,000 dilution of secondary goat anti-Rabbit IgG (H+L) highly cross-adsorbed antibody, Alexa Fluor 555 (Invitrogen) in 1% Roti™ImmunoBlock/PBS.
- If applicable: Prepare Hoechst stain, as explained above.

#### 3.3.2. Staining Procedure

**Figure 7:**
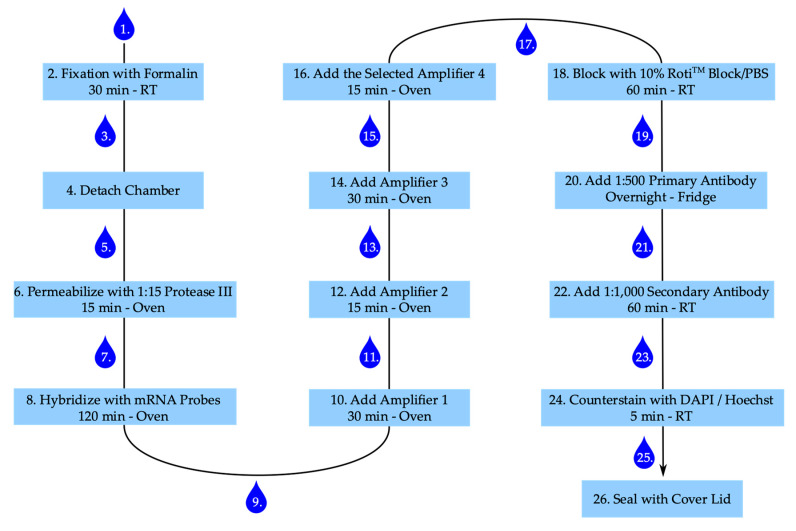
Staining procedure for RNAscope™ and senescence-associated heterochromatin foci. Numbering represents steps in staining procedure. Drop corresponds with washing step. RT: room temperature.

The step-by-step staining procedure according to the manufactures’ recommendations (ACD Bio) is given below. Figure 7 summarizes the main points with several incubation steps, including one overnight incubation time.

1. After stimulation aspirate medium and wash cells once with PBS.
2. Add 300 µL of 4% buffered formalin to each well and fix cells for 30 min at room temperature.
3. Aspirate formalin and wash twice with PBS.
4. Carefully detach the chamber from the slide according to the manufacturer’s instruction and place the slide in a PBS filled washing dish. Caution: The glue is strong. Make sure to remove the glue of the chamber properly, otherwise the slide–coverslip combination becomes too thick.
5. Remove slide from the washing dish and thoroughly apply a barrier around each well with the ImmEdge™ hydrophobic barrier pen (ACD Bio) and place slide again in PBS.
6. Remove the slide from PBS, remove attaching PBS by gently inverting the slide and add 50 µL of diluted Protease III to each well. Place slide in the humidity control tray, close humidity control tray, and incubate in the oven for 15 min.
7. Remove slides from tray; remove protease from slide by inverting the slide and place slide in fresh PBS. Caution: The movement should be gently, but still removing the majority of liquid.
8. Remove slide from PBS, remove PBS by gently inverting the slide, and add 50 µL of diluted target probes or one drop of positive or negative control to the according wells, place slides in the humidity control tray, and incubate in the oven for 120 min.
9. Take slides out of the tray, inverse, and wash twice for 2 min each in wash buffer.
10. Remove attached liquid by gentle inversion and add one drop of amplifier 1-fluid (Amp 1-FL) to each well. Put slides in the humidity control tray in the oven for 30 min.
11. Take slides out of the tray, inverse, and wash twice for 2 min each in wash buffer.
12. Remove attached liquid by gentle inversion and add 1 drop of Amp 2-FL to each well, place in the humidity control tray in the oven for 15 min.
13. After 15 min take slides out of the tray, inverse and wash twice for 2 min each in wash buffer.
14. Remove attached liquid by gentle inversion and add 1 drop of Amp 3-FL to each well, place in the humidity control tray in the oven for 30 min.
15. Take slides out of the tray, inverse and wash twice for 2 min each in wash buffer.
16. Remove attached liquid by gentle inversion and add 1 drop of the selected Amp 4-FL to each well, place in the humidity control tray in the oven for 15 min. Caution: For the multiplexing protocol we used Amp 4-FL A.
17. Take slides out of the tray, inverse and wash twice for 2 min each in wash buffer.
18. Add 100 µL of 10% Roti™ImmunoBlock/PBS to each well and block for 1 h in the closed humidity control tray at room temperature.
19. Remove attached liquid by gentle inversion and wash once with PBS.
20. Add 50 µL 1:500 dilution of primary anti-histone H2A.X (phosphoS139) antibody [EP854(2)Y] (Abcam) in 1% Roti™ImmunoBlock/PBS per well and incubate in the closed humidity control tray in the fridge overnight.
21. Remove attached liquid by gentle inversion and wash twice with PBS.
22. Add 50 µL 1:1,000 dilution of secondary goat anti-rabbit IgG (H+L) Highly Cross-Adsorbed antibody, Alexa Fluor 555 (Invitrogen) in 1% Roti™ImmunoBlock/PBS per well and incubate in the closed humidity control tray at room temperature for 60 min.
23. Remove attached liquid by gentle inversion and wash twice with PBS.
24. Add 1 drop of 4′,6-diamidino-2-phenylindole (DAPI) per well and incubate for 1 min at room temperature (alternatively add 50 µL 1:2,000 Hoechst working solution and incubate for 5 min at room temperature).
25. Remove attached liquid by gentle inversion and wash once with PBS.
26. Add two drops of ProLong™ diamond antifade medium (Thermo Scientific) to each slide and gently apply the lid, make sure to gently remove all bubbles, and harden overnight in the fridge. Caution: The medium is highly viscose and can easily dry out. The cover slid is still moveable, even after drying. Be careful when cleaning the slide for imaging. Sealing the slide-lid combination with nail varnish can help in preventing the drying out and preserving the slides for later imaging.

### 3.4. Imaging

For imaging any fluorescence microscope with appropriate filter setting or a confocal microscope is possible. We obtained the images while using a Zeiss Axiovert 200M inverted transmitted light microscope using the filter sets, as illustrated in Table 1. For image acquisition, the 40× F-Fluar objective with Immersol™ oil and the Zen software (Zeiss, Zen2 blue edition) was used. Each experiment was done in three independent experiments with duplicates for each stimulation drug. An acquisition of five images per well was done for analysis.

### 3.5. Quantification of Fluorescence Intensity

The fluorescence intensity per channel was quantified while using the Zen2 software (Zeiss, blue edition). Five images per well/stimulation were analyzed for three independent experiments.

### 3.6. Statistical Analysis

Mean ± SEM is given in the bar graph. Statistical significance between stimulation and respective control was analyzed using the Mann-Whitney-U Test. A *p* value < 0.05 was set as statistically significant.

## 4. Conclusions

In conclusion, the new experimental sets that are presented here allow for multiplexing and quantification of several markers of interest for calcification and senescence analysis in primary VSMC. This might not only help to reduce animal numbers for primary cell isolation and in vivo settings with regard to the 3R (Replacement, Reduction, and Refinement) thought of Russel and Burch [19], but also allow the visualization of markers of interest on a single cell basis.

## Figures and Tables

**Table 1:** Excitation/Emission Wavelength of the Dyes and Filters Used.

Dye	SPiDER SA-β-Gal	Hoechst 33342	Alexa 488	Alexa 555	Atto 647	DAPI
Beam Splitter	532	395	495	570	660	395
Filter Ex. Wavelength	500–530	335–383	450–490	538–562	625–665	335–383
Filter Em. Wavelength	545–605	420–470	500–550	570–640	665–715	420–470
Ex. Wavelength of Dye	528	348	493	553	644	348
Em. Wavelength of Dye	547	455	517	568	670	455

## Supplementary Materials

Supplementary materials can be found at https://www.mdpi.com/1422-0067/21/10/3475/s1.

## Abbreviations

Amp-FL	Amplifier fluid
Ctrl	Control
DAPI	4′,6-diamidino-2-phenylindole
DMSO	Dimethylsulfoxide
DNA	Deoxyribonucleic acid
DMEM	Dulbeccos-modified Eagle medium
Dox	Doxorubicin
FCS	Fetal calf serum
mRNA	Messenger Ribonucleic acid
OPN	Osteopontin
PCR	Polymerase chain reaction
PBS	Phosphate-buffered saline
RT	Room temperature
rVSMC	Rat vascular smooth muscle cell
SA-β-Gal	Senescence-associated β-galactosidase
SAHF	Senescence-associated heterochromatin foci
SEM	Standard error of mean
Up_4_A	Uridine adenosine tetraphosphate
VSMC	Vascular smooth muscle cell
X-Gal (BCIG)	5-bromo-4-chloro-3-indolyl-β-D-galactopyranoside
