# Supplementary material for: A Novel Protocol for Detection of Senescence and Calcification Markers by Fluorescence Microscopy
Source: Int J Mol Sci. 2020 May 14;21(10):3475. doi: 10.3390/ijms21103475 (PMC7278918; doi:10.3390/ijms21103475)
Supplement: Supplementary file 1 [file ijms-21-03475-s001.pdf]

Supplementary Material

1.1. Comparision of currently available detection methods

The suppl. Table 1 summarizes a comparison of the currently available detection methods for detection and measurement of mRNA or protein expression of mentioned senescence and osteoblastic markers during vascular calcification in cell culture experiments.

Table S1. Summary of pros and cons of currently available detection methods.

Methods	Disadvantages	Advantages
**qPCR**	– Bulk of cells are analyzed jointly, individual differences are not visible in this method<br>– Quantification usually relative	– Multiple target genes can be analyzed<br>– Analysis easily reproducible
**Single Cell PCR**	– Multistep, complex method<br>– Difficult to train and error prone<br>– Versatile hardware required	– Variety of target genes can be analyzed<br>– Cluster analysis provides additional information
**Immunohistochemistry**	– Due to issues of specificity of antibodies often requires extensive protocol establishment<br>– Monoclonal and polyclonal antibodies are of animal origin and can suffer from batch variances<br>– Development of suitable protocol (blocking, pretreatment, etc.) often time and material consuming process<br>– Only Semi-Quantification possible<br>– Limited number of targets<br>– Multiplexing can be complicated, especially if different secondary antibodies are employed	– Procedure with functioning protocol easily manageable, easy to teach and learn<br>– Recombinant antibodies are animal free produced and have less issues with batch variances<br>– Few hardware required<br>– Multiplexing possible
**RNA In Situ Hybridization**	– Depending on the protocol, the number of targets is limited; in the presented protocol, the number of targets that can be analyzed parallelly is	– Different labeling and detection possible (radioactive, fluorescence and immunohisto-chemistry)<br>– Huge variety of targets can

	limited to 3<br>– Extensive hardware required	be analyzed, as probes can be custom designed to targets<br>– Single cell analysis in the tissue context is possible<br>– Multiple target genes can be analyzed at once<br>– Small growth area reduces necessary number of cells, working hours and material
**X-Gal**	– Long incubation time<br>– Process of staining vulnerable to external influences (e.g., pH variation due to long staining times)<br>– Semi-Quantification difficult<br>– No Multiplexing	– Cheap<br>– Frequently published protocol<br>– Little hardware required<br>–
**SPiDER™ ß Gal Stain**	– Multiplexing is difficult, as permeabilization of cells usually required for multiplexing facilitates washout of stain<br>– Extensive hardware required	– Short and easy staining<br>– Semi-Quantification possible, as image is easy to visualize<br>– Multiplexing is possible

1.2. Reagents for cell stimulation

Table S2. Reagents for cell stimulation

Material	Company	Order Number
**Doxorubicin hydrochloride**	Thermo Fisher	BP2516-10
**Up_4A**	Jena Bioscience	NU-528S

1.3. Kits and antibodies for fluorescence detection

Table S3. Kits and antibodies for fluorescence detection

Material	Company	Order Number
**SPiDER™**	Gerbu Biotechnologie	SG02-10
**RNAscope™ Target Retrieval Reagents Containing: Protease III**	ACD Bio	322000
**RNAscope™ Fluorescent Multiplex Reagent Kit Containing: Amp 1-FL**	ACD Bio	320850

**Amp 2-FL<br>Amp 3-FL<br>Amp 4-FL<br>DAPI**		
**RNAscope™ Wash Buffer Reagents**	ACD Bio	310091
**ImmEdge™ Hydrophobic Barrier Pen**	ACD Bio	310018
**RNAscope™ Probe - Rn-Cdkn1a**	ACD Bio	423851-C3
**RNAscope™ Probe - Rn-Spp1**	ACD Bio	405441
**RNAscope™ 3-plex Positive Control Probe - Rn**	ACD Bio	320891
**RNAscope™ 3-plex Negative Control Probe - Rn**	ACD Bio	320871
**Anti-Histone H2A.X (phospho S139) antibody [EP854(2)Y]**	abcam	ab215967
**Goat anti-Rabbit IgG (H+L) Highly Cross-Adsorbed Secondary Antibody, Alexa Fluor 555**	Invitrogen	A-21429
**ProLong™ Diamond antifade medium**	Thermo Fisher Scientific	P10144
